# The One Health Approach—Why Is It So Important?

**DOI:** 10.3390/tropicalmed4020088

**Published:** 2019-05-31

**Authors:** John S Mackenzie, Martyn Jeggo

**Affiliations:** 1 PathWest, Queen Elizabeth 2 Medical Centre, Nedlands, WA 6009, Australia; 2 Faculty of Health Sciences, Curtin University, GPO Box U1987, Perth, WA 6845, Australia; 3 One Health Platform Foundation, Overheet 48, 9290 Berlare, Belgium; 4 AUSGEM Governing Board, 31 The Breakwater, Corlette, NSW 2315, Australia; jeggo.martyn@gmail.com

It has become increasingly clear over the past three decades that the majority of novel, emergent zoonotic infectious diseases originate in animals, especially wildlife [1], and that the principal drivers of their emergence are associated with human activities, including changes in ecosystems and land use, intensification of agriculture, urbanisation, and international travel and trade [2,3,4,5,6]. A collaborative and multi-disciplinary approach, cutting across boundaries of animal, human, and environmental health, is needed to understand the ecology of each emerging zoonotic disease in order to undertake a risk assessment, and to develop plans for response and control.

The term ‘One Health’ was first used in 2003–2004, and was associated with the emergence of severe acute respiratory disease (SARS) in early 2003 and subsequently by the spread of highly pathogenic avian influenza H5N1, and by the series of strategic goals known as the ‘Manhattan Principles’ derived at a meeting of the Wildlife Conservation Society in 2004, which clearly recognised the link between human and animal health and the threats that diseases pose to food supplies and economies. These principles were a vital step in recognising the critical importance of collaborative, cross-disciplinary approaches for responding to emerging and resurging diseases, and in particular, for the inclusion of wildlife health as an essential component of global disease prevention, surveillance, control, and mitigation [7].

The outbreak of SARS, the first severe and readily transmissible novel disease to emerge in the 21st century, led to the realisation that (a) a previously unknown pathogen could emerge from a wildlife source at any time and in any place and, without warning, threaten the health, well-being, and economies of all societies; (b) there was a clear need for countries to have the capability and capacity to maintain an effective alert and response system to detect and quickly react to outbreaks of international concern, and to share information about such outbreaks rapidly and transparently; and (c) responding to large multi-country outbreaks or pandemics requires global cooperation and global participation using the basic principles enshrined in One Health [8]. The emergence and spread of influenza H5N1 has been another excellent example of the importance of global cooperation and a One Health approach driven by the widespread concern that it might become the next influenza pandemic strain. It also served as a catalyst for the United Nations Secretary General to appoint a UN Systems Coordinator for Avian and Animal Influenza (UNSIC), and to form a major collaboration with a number of international and national organizations, including the World Health Organization (WHO), Food and Agriculture Organization (FAO), World Organization for Animal Health (OIE), United Nations Children’s Fund (UNICEF), and World Bank and various national heath ministries, to develop the International Ministerial Conferences on Avian and Pandemic Influenza (IMCAPI). IMCAPI was a major driver in the surveillance and responses to influenza H5N1 [9] and subsequently in the development of a strategic framework built around a One Health approach that focussed on diminishing the risk and minimizing the global impact of epidemics and pandemics due to emerging infectious diseases [10].

The concept of One Health is not new and can be traced back for at least two hundred years [11], firstly as One Medicine, but then as One World, One Health and eventually One Health. There is no single, internationally agreed upon definition of One Health, although several have been suggested. The most commonly used definition shared by the US Centers for Disease Control and Prevention and the One Health Commission is: ‘One Health is defined as a collaborative, multisectoral, and transdisciplinary approach—working at the local, regional, national, and global levels—with the goal of achieving optimal health outcomes recognizing the interconnection between people, animals, plants, and their shared environment’. A definition suggested by the One Health Global Network is: ‘One Health recognizes that the health of humans, animals and ecosystems are interconnected. It involves applying a coordinated, collaborative, multidisciplinary and cross-sectoral approach to address potential or existing risks that originate at the animal-human-ecosystems interface’. A much simpler version of these two definitions is provided by the One Health Institute of the University of California at Davis: ‘One Health is an approach to ensure the well-being of people, animals and the environment through collaborative problem solving—locally, nationally, and globally’. Others have a much broader view, as encapsulated in Figure 1.

The One Health concept clearly focusses on consequences, responses, and actions at the animal–human–ecosystems interfaces, and especially (a) emerging and endemic zoonoses, the latter being responsible for a much greater burden of disease in the developing world, with a major societal impact in resource-poor settings [12,13]; antimicrobial resistance (AMR), as resistance can arise in humans, animals, or the environment, and may spread from one to the other, and from one country to another [14,15,16,17]; and food safety [18,19]. However, the scope of One Health as envisaged by the international organizations (WHO, FAO, OIE, UNICEF), the World Bank, and many national organisations also clearly embraces other disciplines and domains, including environmental and ecosystem health, social sciences, ecology, wildlife, land use, and biodiversity. Interdisciplinary collaboration is at the heart of the One Health concept, but while the veterinarian community has embraced the One Health concept, the medical community has been much slower to fully engage, despite support for One Health from bodies such as the American Medical Association, Public Health England, and WHO. Engaging the medical community more fully in the future may require the incorporation of the One Health concept into the medical school curricula so that medical students see it as an essential component in the context of public health and infectious diseases [20].

One recent development that might help in generating increased global awareness of the One Health concept, particularly among students, but also more generally, has been the designation of November 3rd as One Health Day. Initiated in 2016 by the One Health Commission (www.onehealthcommission.org), the One Health Platform Foundation (www.onehealthplatform.com), and the One Health Initiative (http://www.onehealthinitiative.com), One Health Day is celebrated through One Health educational and awareness events held around the world. Students are especially encouraged to envision and implement One Health projects, and to enter them into an annual competition for the best student-led initiatives in each of four global regions.

Today’s health problems are frequently complex, transboundary, multifactorial, and across species, and if approached from a purely medical, veterinary, or ecological standpoint, it is unlikely that sustainable mitigation strategies will be produced.

This special issue of *Tropical Medicine and Infectious Disease* contains a series of papers taking a One Health approach to a range of infectious diseases and the broader topic of antimicrobial resistance at the animal–human–environment interface, as well as to aspects of policy concerned with trade issues relating to AMR in the food chain and with aspects of public health policy and practice where significant knowledge gaps in the translation of scientific expertise and results, and biosafety and biosecurity measures, need to be addressed. These examples illustrate the critical importance of using a One Health approach for understanding and mitigating many current complex health problems. They demonstrate not only innovative approaches and outcomes but the range and types of collaborative partnerships that are required. This collection of papers demonstrates the breadth and scope of One Health, partly from an Australasian perspective, but also with an international flavour. They also serve to demonstrate the critical importance of taking a One Health approach to problems that have defied a more traditional disciplinary or sectoral approach.

## Figures and Tables

**Figure 1:**
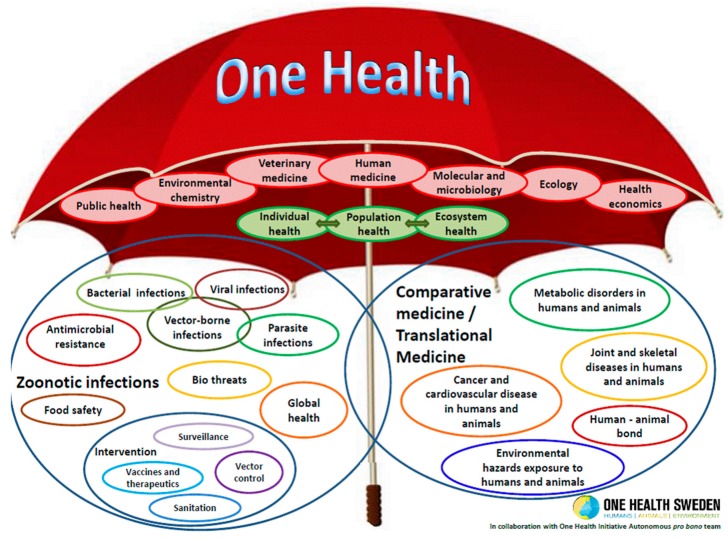
The One Health Umbrella, developed by One Health Sweden and the One Health Initiative Autonomous pro bono team.
